# Accurate Measurements of Wall Shear Stress on a Plate with Elliptic Leading Edge

**DOI:** 10.3390/s18082682

**Published:** 2018-08-15

**Authors:** Guang-Hui Ding, Bing-He Ma, Jin-Jun Deng, Wei-Zheng Yuan, Kang Liu

**Affiliations:** Key Laboratory of Micro/Nano Systems for Aerospace, Ministry of Education, Northwestern Polytechnical University, Xi’an 710072, China; 2015100434@mail.nwpu.edu.cn (G.-H.D.); dengjj@nwpu.edu.cn (J.-J.D.); yuanwz@nwpu.edu.cn (W.-Z.Y.); liukang2016@mail.nwpu.edu.cn (K.L.)

**Keywords:** wall shear stress, MEMS, floating element, turbulent boundary layer, smooth plate

## Abstract

A micro-floating element wall shear stress sensor with backside connections has been developed for accurate measurements of wall shear stress under the turbulent boundary layer. The micro-sensor was designed and fabricated on a 10.16 cm SOI (Silicon on Insulator) wafer by MEMS (Micro-Electro-Mechanical System) processing technology. Then, it was calibrated by a wind tunnel setup over a range of 0 Pa to 65 Pa. The measurements of wall shear stress on a smooth plate were carried out in a 0.6 m × 0.6 m transonic wind tunnel. Flow speed ranges from 0.4 Ma to 0.8 Ma, with a corresponding Reynold number of 1.05 × 10^6^~1.55 × 10^6^ at the micro-sensor location. Wall shear stress measured by the micro-sensor has a range of about 34 Pa to 93 Pa, which is consistent with theoretical values. For comparisons, a Preston tube was also used to measure wall shear stress at the same time. The results show that wall shear stress obtained by three methods (the micro-sensor, a Preston tube, and theoretical results) are well agreed with each other.

## 1. Introduction

Accurate measurements of wall shear stress are crucial for judging flow phenomena in the laminar/turbulent boundary layer [1,2]. For example, the values of wall shear stress become more fluctuating when flow transition occurs, then it will decrease sharply at the point where there is flow separation. Generally, the magnitude of wall shear stress in the turbulent boundary layer is larger than that in the laminar boundary layer. Therefore, eliminating or delaying flow transition position on airplane wings is helpful to reduces viscous drag during the flight, which is a key point for energy-saving travels. Also, aerodynamic performance of aircrafts can be well evaluated by pre-knowledge of accurate distributions of wall shear stress and wall pressure. Wall shear stress also plays an important role in verifying or revising turbulent model. Accurate measurements of wall shear stress give a special view of flow phenomena under the boundary. It can be used to evaluating flow fluctuations, developments of turbulent boundary layer, and so on. For now, wall shear stress can be measured by Preston tubes [3], hot wires/films [4,5], micro-pillars [6,7], sub-layer fences [8], and micro-floating element wall shear stress sensors [9,10,11,12,13,14,15]. These techniques have been developed for many years and have been proved a good prospect in aerodynamic measurements. Advantages and disadvantages have been discussed in plenty of literatures [16,17]. Some researchers think direct measurements of wall shear stress are preferred because no assumptions of flow conditions are required [18]. Given such the point, MEMS-based floating element wall shear stress sensors have shown a good potential for various aerodynamic applications.

In this paper, we present a micro-machined floating element wall shear stress sensor with capacitive sensing. The micro-sensor is fabricated on a 10.16 cm SOI wafer and it has backside electrodes for non-invasive measurements. The packaged wall shear stress sensor is calibrated over a range of 0 Pa to 65 Pa, then it is used to measure wall shear stress on a smooth plate with elliptic leading edge. For comparisons, a Preston tube is located near the sensor position. The results obtained by the floating element wall shear stress sensor and the Preston tube agree well.

## 2. Micro-Floating Element Wall Shear Stress Sensor

### 2.1. Sensor Structures

**Figure 1:**
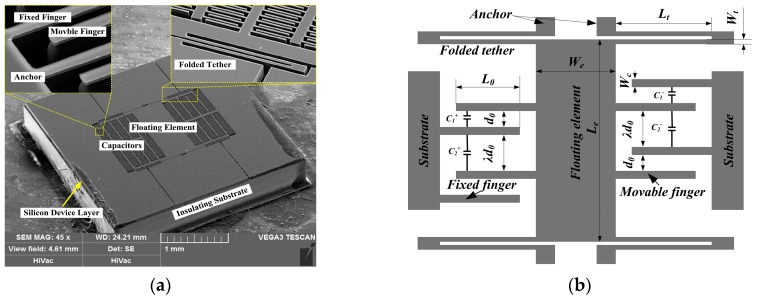
(**a**) shows SEM images of the floating element wall shear stress sensor with capacitive sensing, and (**b**) gives descriptions of the sensors’ parameters.

The micro-floating element wall shear stress sensor consists of a floating element, four folded beams, a number of comb fingers, and some anchors, as shown in Figure 1a. Differential capacitors are designed for improving the sensitivity of the micro-sensor and lowing noise. Compared to clamped-clamped beams, folded beams have a larger linear displacement and they are helpful to release structural stress along the lateral direction of the sensor die. When the sensor is exposed to airflow, the floating element moves a micro displacement along the flow direction, changing the gaps between neighboring comb fingers. Therefore, the applied wall shear stress can be referred by the capacitance difference of the differential capacitors.

According to Euler–Bernoulli beam theory, the total stiffness of the wall shear stress sensor is expressed as:

(1) $$k=2Et{\left(W_{t}/L_{t}\right)}^{3},$$

where *E (E_100_ =* 130 GPa in this study) is Young’s modulus of Silicon; *t*, *W_t_*, and *L_t_* are the thickness, width, and length of folded beams, respectively. Wall shear stress applied to the folded beams can be ignored because it has small effects on the floating element’s motion. Therefore, displacement of the floating element (*δ*) under a known wall shear stress (*τ_w_*) is given as:

(2) $$\delta \;=F/k\;=\;[\tau_{w}(W_{e}L_{e}+NW_{c}L_{c})]/[2Et{\left(W_{t}/L_{t}\right)}^{3}],$$

where *W_e_* and *L_e_* are the width and length of floating element, respectively. *W_c_*, *L_c_*, and *N* are the width, length, and the number of movable comb fingers, respectively. As a second order sensor, the floating element wall shear stress sensor can be equivalent to a spring-mass system with low damping ratio. When we ignore the mass of the folded tether, the resonance frequency of the floating element can be expressed as:

(3) $$f_{0}=\frac{1}{2\pi }\sqrt{\frac{k}{m}}=\frac{1}{2\pi }\sqrt{\frac{2E}{\rho \left(W_{e}L_{e}+NW_{c}L_{c}\right)}{\left(\frac{W_{t}}{L_{t}}\right)}^{3}},$$

where *m* the mass of movable structures, and *ρ* is the density of Silicon. Generally, the response frequency of the sensor is about 1/5–1/3 of its resonance frequency. However, higher mechanical resonance usually results in lower sensitivity of the sensor, and the tradeoff between them should be carefully considered under the specific applications.

The differential capacitors are settled on two sides of the floating element and the parameters are shown in Figure 1b. When the floating element moves a micro displacement (*δ*) along the direction of flow, the capacitance difference of the sensor die can be expressed as [15]:

(4) $$\Delta C=C^{+}-C^{-}=\frac{2\varepsilon NL_{0}t\delta }{d_{0}{}^{2}-\delta^{2}}-\frac{2\varepsilon NL_{0}t\delta }{\lambda^{2}d_{0}{}^{2}-\delta^{2}},$$

where *ε* is the permittivity of air, *L*_0_ is the overlapping length of comb fingers, *d*_0_ is the initial gaps between neighboring comb fingers, and *λ* is the gaps amplification factor of comb fingers. When the displacement of floating element is far less than the initial gaps between comb fingers (*δ* *<< d*_0_), expressing (4) in Taylor Expansions form and then ignore the high order terms, then we can get:

(5) $$\Delta C^{\sim }=\frac{2\varepsilon NL_{0}t}{d_{0}{}^{2}}\left(1-\frac{1}{\lambda^{2}}\right)\delta .$$

However, the approximate expression (5) represents the nonlinear output of the sensor die when the displacement of the floating element changes. Thus, we can obtain the nonlinearity of the sensor according to (4) and (5) as:

(6) $$\gamma =\left|\frac{\Delta C-\Delta C^{\sim }}{\Delta C}\right|\times 100\%=\left|{\left[1-{\left(\delta /d_{0}\right)}^{2}\right]}^{-1}{\left\{1-{\left[\delta /\left(\lambda d_{0}\right)\right]}^{2}\right\}}^{-1}-1\right|\times 100\%.$$

In (6), the nonlinearity of the sensor is related to the displacement of the floating element. In our design, the gaps amplification factor is designed as *λ* = 2.5, thus the nonlinearity of the sensor will be about 1.17% when *δ* = 0.1 × *d*_0_. 

Robert White’s group’s research shows that the sensitivity to wall shear stress is much larger than that to pressure gradient [11]. Generally, assuming that pressure gradient is two orders larger than wall shear stress, the contribution from pressure gradient to the sensor output is less than 1% of that from wall shear stress. Thus, the sensitivity of the sensor is described as the first derivative of the capacitance difference versus applied wall shear stress,

(7) $$S=\frac{\partial \Delta C^{\sim }}{\partial \tau_{w}}=\frac{\partial \Delta C^{\sim }}{\partial \delta }\frac{\partial \delta }{\partial \tau_{w}}=\frac{\left(W_{e}L_{e}+NW_{c}L_{c}\right)\varepsilon NL_{0}}{Ed_{0}{}^{2}{\left(W_{t}/L_{t}\right)}^{3}}\left(1-\frac{1}{\lambda^{2}}\right).$$

### 2.2. Fabrication Process

**Figure 2:**
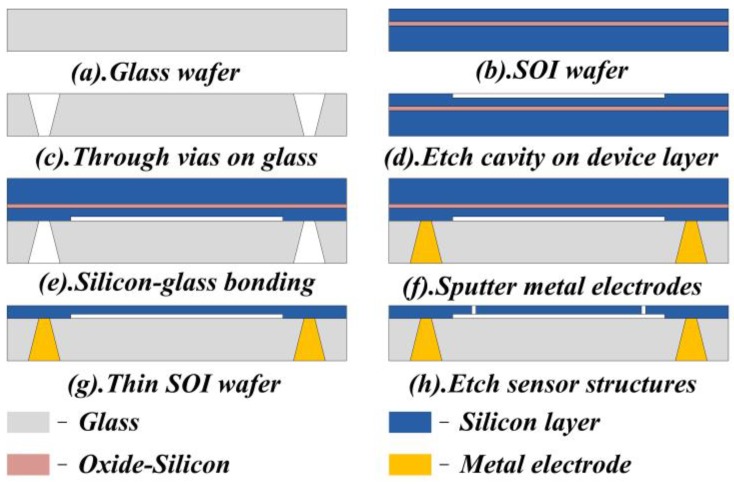
Fabrication process of the micro-floating element wall shear stress sensor with backside connections.

Main structures of the floating element wall shear stress sensor in this study are micro-machined on the device layer of a 10.16 cm SOI wafer. Then a 10.16 cm Borofloat33 glass wafer is employed to support the silicon structures of the sensor. The SOI wafer has a device layer of 50 μm in thickness and the fabrication process is shown in Figure 2. 

**Figure 3:**
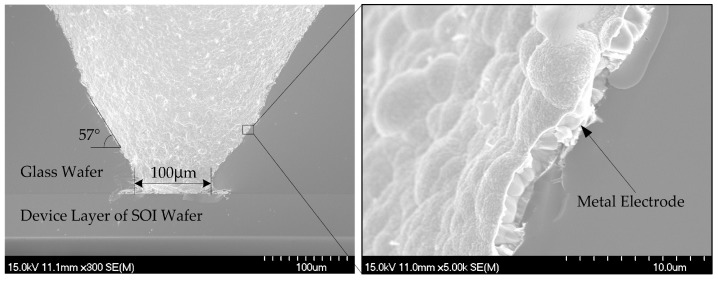
SEM images of the conical through vias on the glass with metal electrodes and their enlarged views.

First of all, a rectangular cavity of 2000 μm × 1200 μm in top-view size is patterned on the device layer of the SOI wafer by photolithography process. Then, the cavity of 5 μm in depth is etched by Inductively Coupled Plasma (ICP) process. Secondly, conical through vias are machined on the glass wafer (300 μm in thickness) in order to form the tunnel for backside electrodes. The conical vias have a smaller end of 100 μm in diameter and a larger end of 500 μm in diameter, respectively. Thus, we can obtain the sloping sidewalls of vias and make it easier to sputter metals onto the sidewalls. Thirdly, the device layer of SOI wafer and the Borofloat33 glass wafer mentioned above are bonded together by anodic bonding process, as shown in Figure 2e. Thus, the cavity will be embedded between the SOI wafer and the glass wafer. After that, 30 nm Ni, 50 nm Cr, and 200 nm Au are sputtered on the SOI wafer and the side walls of glass vias as backside electrodes. Ni and Cr layers are used to strengthen adhesion between the Au layer and the sensor die. The sidewalls have a slope angle of about 57° and it helps to deposit metal layers on surface of the sidewalls. Figure 3 shows the SEM images of the conical through vias on the glass wafer with metal electrodes. Then the substrate layer and the oxide layer of the SOI wafer are removed for the following process. Another photolithography process is required for the sensor structures’ pattern. Finally, the sensor structures are micro-machined and released by ICP etching process.

Actually, unavoidable machining errors during fabrication process are responsible for poor performance of the micro-sensor. For example, sensor structures will be over etched during the ICP etching process, which is harmful to sensor’s measurement ranges. Therefore, the sensor performance should be evaluated by measured parameters, rather than the designed ones. The structural parameters of the floating element wall shear stress sensor are shown in Table 1.

According to (2), when the displacement of the floating element equals to 0.41 μm, the relevant wall shear stress can be calculated as *τ_w_* = 107 Pa. Then, the nonlinearity of the sensor is calculated as *γ* = 1.26% by (6). The resonance frequency of the sensor calculated by (3) is about 7.5 kHz.

### 2.3. Interface Circuit and Package

**Figure 4:**
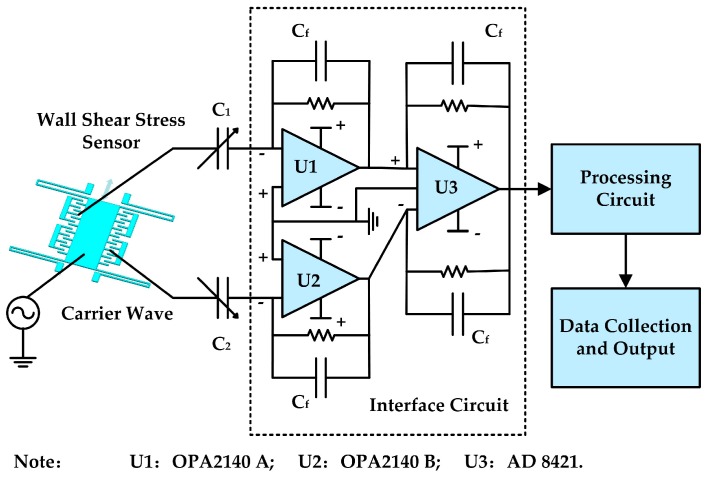
Interface circuit of the floating element wall shear stress sensor.

Figure 4 illustrates the interface circuit of the micro-floating element wall shear stress sensor with differential capacitive sensing. A fully differential charge amplifier circuit is used for detecting the capacitance of the sensor die. It extracts the sensor’s high-impedance output signal as a charge, and then the charge is gained, converted to a voltage, and buffered for transmission. In this study, the 1 Vpp, 439 kHz sinusoidal carrier wave is applied to common electrode of the sensor die, and the differential capacitors are connected to two input pins of an OPA 2140 (U1 and U2) chip for capacitance-to-voltage (C–V) conversions, respectively. Then the outputs of the OPA 2140 chip are transferred to an AD 8421 chip for difference processing. Reference capacitance and reference resistance are 2.5 pF and 100 MΩ, respectively. Finally, the output of AD 8421 chip is demodulated and filtered by the post-processing circuit and then the data are obtained by a 16-bit DAQ (NI USB-6120).

**Figure 5:**
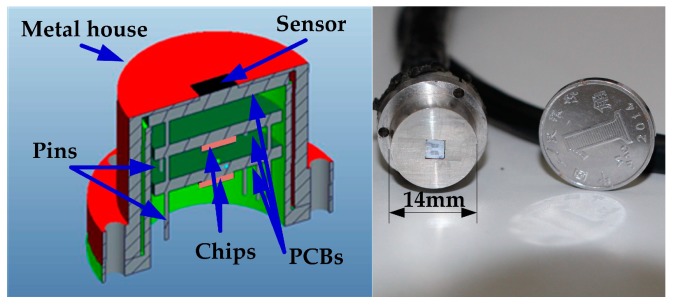
Package of the micro-sensor with backside connections and the picture of packaged sensor probe.

Package of the sensor is critical for its practical applications. From structural parameters from Table 1, the initial capacitance of the sensor die is about 3 pF, which is easily affected by parasitic capacitance from connecting wires. It benefits a lot if the sensor die is put close to the interface circuit. In order to miniaturize the package size, three printed circuit board (PCB) layers are designed to complete the interface circuit. The sensor die, OPA 2140 chip, and AD 8421 chip are placed on the first, second, and third PCB layer, respectively. Then they are packaged in a stainless steel house, which has a square hole to ensure the sensor die is exposed to airflow, shown in Figure 5.

The outer diameter of the package house is 14 mm and the height above the shoulder is 7 mm. Three through holes (2.5 mm in diameter) are made on the shoulder of the package to accomplish the sensor’s installation. The package house is machined with a high precision to ensure the sensor can be flush-mounted on the plate.

## 3. Static Calibration of the Micro-Sensor

**Figure 6:**
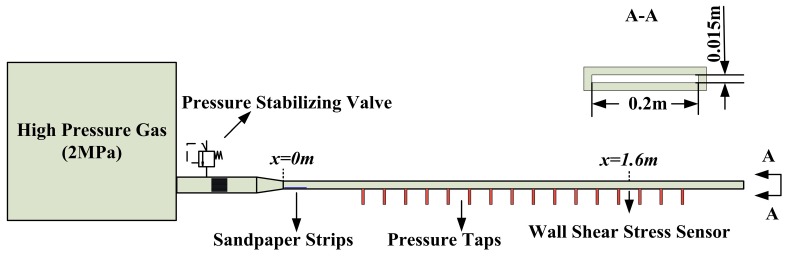
The wind tunnel setup for static calibration of the micro-sensor.

The floating element wall shear stress sensor is calibrated by a high-speed wind tunnel setup, which is located in China Aerodynamics Research and Development Center (CARDC). The wind tunnel setup consists of a high-pressure air source, a pressure stabilizing valve, a wind tunnel (2 m in length and 0.2 m × 0.015 m in size of the cross section), and some pressure holes distributed on the bottom of the wind tunnel, as shown in Figure 6.

**Figure 7:**
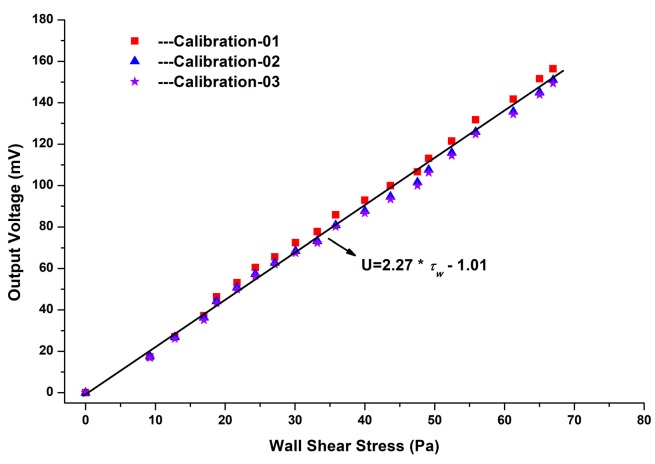
Calibration results of the micro-floating element wall shear stress sensor.

The packaged sensor probe and a reference Preston tube are flush-mounted on the opposite sides of the tunnel at the same axial location (*x* = 1.6 m) for the equal wall shear stress. Flow speed is well controlled by the pressure stabilizing valve and the maximum Mach number can reach to about 0.67 Ma, which is corresponding to wall shear stress of about 65 Pa. The flow speed below the Mach number of 0.3 is considered as incompressible and the flow conditions are not steady in the wind tunnel. Therefore, the sensor is calibrated over a Mach number range of 0.3 to 0.65. The corresponding Reynold number ranges from about 7.4 × 10^4^ to 2.6 × 10^5^ and the flow conditions of the turbulent boundary layer has been fully developed at the sensor position (*x* = 1.6 m). Therefore, wall shear stress is given by pressure gradients along the wind tunnel. The output data of the sensor are fitted in Figure 7.

It is found the calibration curves are straight lines because that the output of the sensor is proportional to the applied wall shear stress. The function of the fitting line is appropriately expressed as:

(8) $$y\;=\;2.27\times \tau_{w}-\;1.01.$$

where y represents the output voltage of the micro-sensor, and *τ_w_* is wall shear stress that applies to the micro-sensor. According to the calibration results, the nonlinearity of the micro-sensor is about 2.9%, and the maximum repeatability error is about 4.7% at *τ_w_* = 67 Pa.

## 4. Experiments and Results

### 4.1. Plate Model and Flow Condtions

**Figure 8:**
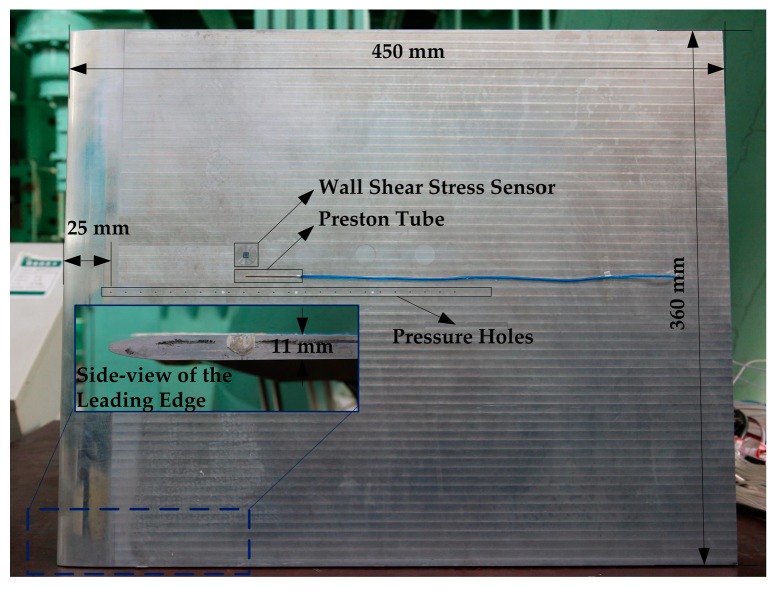
Picture of the plate model with an elliptic leading edge and the sensor installation.

A smooth plate model with elliptic leading edge is designed and fabricated for wall shear stress measurements in this study. The rigid plate has a length of 450 mm, a width of 360 mm, and a thickness of 11 mm, as shown in Figure 8. The governing equation of the elliptic leading edge is expressed as:

(9) $${\left(\frac{a}{33}\right)}^{3.2}+{\left(\frac{b}{5.5}\right)}^{3.2}=1.$$

Along the longitudinal direction of the plate, 24 pressure holes (0.7 mm in diameter) are drilled at the plate’s central line and the distance of neighboring holes is 10 mm. The floating element wall shear sensor and a Preston tube are flush-mounted on the plate and the distance between the sensor and the leading edge is 124 mm. Then, they were mounted in a transonic wind tunnel with the total pressure of airflow is about 93 kPa. Wall temperature in this experiment ranges from about 285 K to 289 K, which is measured by a commercial thermocouple located on the plate. We assume that the plate surface is adiabatic and the can obtain the static temperature of airflow by:

(8) $$T_{s}=T_{w}/\left(1+\sqrt{\mathrm{Pr}}\times \frac{\gamma -1}{2}\times Ma^{2}\right),$$

where Pr is Prandtl number, γ is the ratio of specific heat capacities of air, and *Ma* is the Mach number of mainstream in the wind tunnel. The Mach number in this study ranges from 0.4 Ma to 0.8 Ma, which is corresponding to a Reynold number of 1.05 × 10^6^~1.55 × 10^6^. Wall shear stress under the turbulent boundary layer on the plate can be expressed as:

(9) τw=0.0296ρairU2(μρairUx)1/5=0.0296ρairU2/Rex1/5,

where *ρ_air_* is the density of air, *U* is the speed of mainstream, and Re*_x_* is the Reynold number at the location of *x =* 0.124 mm on the plate. According to (8), wall shear stress ranges from about 34.79 Pa to about 91.80 Pa with respect to a Mach number ranging from 0.4 Ma to 0.8 Ma.

### 4.2. Results and Discussion

**Figure 9:**
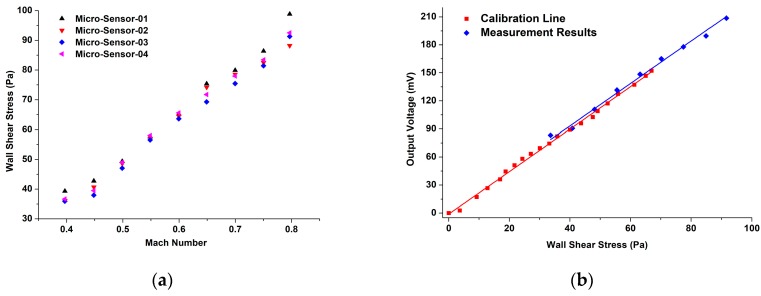
Wall shear stress obtained by the micro-sensor: (**a**) Three repetitive results of wall shear stress on the plate; (**b**) Comparison of the sensor calibration results and prediction results of wall shear stress by the sensor.

Wall shear stress was obtained by three ways in this study: theoretical calculation, a Preston tube, and the floating element wall shear stress sensor. Figure 9a gives four repetitive results obtained by the micro-sensor. The measured wall shear stress ranges from about 35 Pa to about 93 Pa, with a Mach number range of 0.4 Ma to 0.8 Ma. We can see that the repeatability errors of wall shear stress in Mach number of 0.4 Ma, 0.45 Ma, and 0.65 Ma to 0.8 Ma are larger than that in Mach number of 0.5 Ma to 0.6 Ma. The reasons can be explained as that the flow conditions in Mach number of 0.5 Ma to 0.6 Ma are steadier.

In order to eliminate the random errors between different measurements, we make an average of the four results and compare the average values with the calibration results of the micro-sensor. In Figure 9b, the red fitting line gives the static property of the calibrated micro-sensor, which is used to predict wall shear stress on the plate. The blue fitting line comes from the average wall shear stress of the four different measurements in Figure 9a. According to the results, we can obtain that the maximum repeatability error is about 8.9%, which occurs at 0.8 Ma. As we can see, the trends of the two fitting lines in Figure 9b are agreed very well, and it indicates that the calibrated micro-sensor can give good predictions of wall shear stress in our measurements.

**Figure 10:**
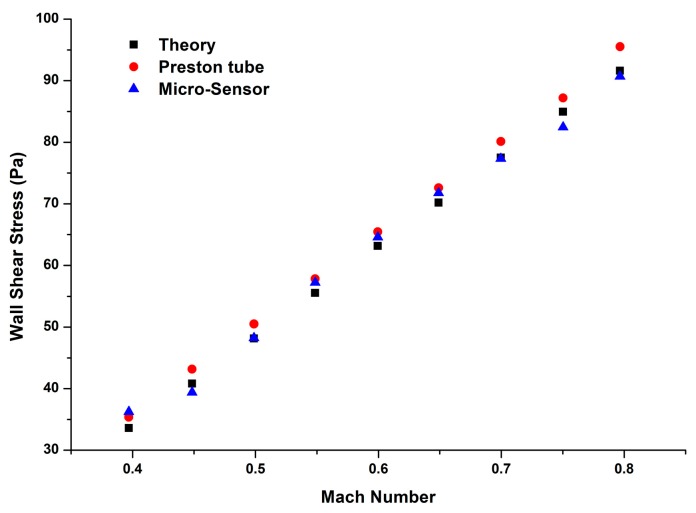
Comparisons of wall shear stress obtained by (9), the Preston tube, and the micro-sensor.

For comparison, wall shear stress is also obtained by a Preston tube, which is located next to the micro-floating element wall shear stress sensor. In Figure 10, the black squares show calculated wall shear stress by (9), and the red circles and blue triangles represent the wall shear stress measured by the Preston tube and the micro-sensor, respectively. Compared to the theoretical wall shear stress, the maximum errors in the Preston tube results and the micro-sensor results are 6.76% and 4.57%, respectively. With the increase of Mach number, the difference between the theoretical values and the Preston tube results becomes larger, which would result in greater errors in measurements. Fortunately, the results obtained by the micro-sensor agree much better with the theoretical predictions.

## 5. Conclusions

A micro-floating element wall shear stress sensor with capacitive sensing has been developed for accurate measurements of wall shear stress under the high-speed turbulent boundary layer. In this study, the micro-sensor was calibrated by a wind tunnel setup over a wall shear stress range of 0 Pa to 65 Pa, and the micro-sensor has a linear output with a nonlinearity of about 2.9%. Accurate measurements of wall shear stress on a smooth plate with an elliptic leading edge have been accomplished in a transonic wind tunnel. The Mach number of airflow ranges from 0.4 Ma to 0.8 Ma with a corresponding Reynold number of 1.05 × 10^6^~1.55 × 10^6^. According to the results, we can obtain the following conclusions:

(1) The micro-floating element wall shear stress sensor is capable of measuring the accurate wall shear stress under the turbulent boundary layer. Backside connections of the micro-sensor enable non-invasive measurements and keep the measured flow continuous and uninterrupted. Compared to indirect methods, hot film for example, it is less sensitive to temperature variations. Therefore, the floating element wall shear stress sensor tends to obtain quantitative results with a higher precision.
(2) Results of wall shear stress on the plate model obtained by the floating element wall shear stress sensor are well agreed with both theoretical results and Preston tube results. Preston tube has been proven as an effective method of measuring mean wall shear stress under the turbulent boundary layer. However, a high response frequency of the sensor is required when measuring wall shear stress under the fluctuating airflow.
(3) For further research, higher Mach number airflow should be investigated to verify the potential applications of the micro-floating element wall shear stress sensor. The micro-sensor can be designed with a high resonance frequency of about 10 kHz, which is helpful to observe the development of the turbulent boundary layer and its fluctuations.

## Figures and Tables

**Table 1:** Structural parameters of the micro-sensor in this study.

Sensor Parameters	Designed Value/μm	Measured Value/μm
Width of the floating element/*W_e_*	680	678.4
Length of the floating element/*L_e_*	1180	1178.2
Width of the folded beams/*W_t_*	10	9.2
Length of the folded beams/*L_t_*	362	360.5
Width of the comb fingers /*W_c_*	5	4.7
Length of the comb fingers/*L_c_*	100	102
Overlapping length of comb fingers/*L*_0_	90	90
Thickness of sensor structures/*t*	45	45
Number of movable comb fingers/*N*	237	237
Initial gap of neighboring comb fingers/*d*_0_	3.0	4.1
Gap amplification factor/*λ*	2.5	2.0
